# The effect of lithium on the structure and function of the human retina: a systematic review

**DOI:** 10.1186/s12886-026-05095-y

**Published:** 2026-07-29

**Authors:** Nicole Needham, David Grosset, Joel T. Martin, Tom MacGillivray, Baljean Dhillon, Jasna Martinovic, Daniel J. Smith

**Affiliations:** 1 School of Neurological and Cardiovascular Sciences, University of Edinburgh, Edinburgh, Scotland; 2 NHS Lothian, Edinburgh, Scotland; 3 School of Philosophy, Psychology and Language Sciences, University of Edinburgh, Edinburgh, Scotland

**Keywords:** Lithium, Retina, Optical coherence tomography, Electrooculography, Electroretinography, Dark adaptation thresholds

## Abstract

**Background:**

Bipolar disorder and depression are associated with structural and functional changes in the retina, including a thinner retinal nerve fibre layer (RNFL). Lithium is widely considered the most effective treatment for bipolar disorder, but its mechanism of action is not fully understood. We assessed research looking at the effect of lithium on structural or functional retinal outcomes in humans.

**Methods:**

Searches using the terms ‘Lithium’ AND ‘retina’ were carried out to identify peer reviewed studies assessing the impact of lithium on retinal structure or function. These included those with or without a control group comparison, pre- and post- lithium comparisons and observational studies. There were no exclusions based on the quantity or preparation of lithium administered, or the length of administration. Risk of bias was assessed using the Joanna Briggs Institute (JBI) critical appraisal tool for Analytical Cross Sectional Studies, and a narrative synthesis and tabulated summary of the included studies was completed.

**Results:**

Seven studies assessing structural outcomes and 10 reporting functional ones were identified, all highly heterogenous and with multiple limitations. Structural outcomes were derived exclusively from optical coherence tomography (OCT) with retinal nerve fibre layer (RNFL) being the most common measurement. There was no evidence of differences in the RNFL between participants with bipolar disorder taking lithium and healthy controls in two larger studies. In six studies looking at differences in those with bipolar disorder taking lithium and those taking valproate, two showed no signs of difference and four showed evidence of thicker RNFL in the lithium group. Studies reporting functional outcomes reported a statistically significant effect of lithium on at least one functional measure, derived from electrooculography, electroretinography, and dark adaptation thresholds.

**Conclusions:**

Current evidence suggests that lithium is likely to have an effect on the retina but limitations in all studies mean better designed and adequately powered prospective studies are required.

**Registration:**

PROSPERO database (Number-CRD42024516635).

**Supplementary Information:**

The online version contains supplementary material available at 10.1186/s12886-026-05095-y.

## Introduction

Lithium is the gold standard treatment for bipolar disorder [1], a lifelong affective disorder affecting 1–2% of the population [2], and is an effective intervention for treatment resistant depression [3]. It has complex neurobiological effects [4], and though its precise therapeutic mechanism is not fully understood, it is known to accumulate in the eye [5, 6], decrease the long- term risk of dementia [7], and influence the circadian rhythm of plants and animals [8]. Chronic lithium administration has been shown to increase retinal lithium levels and decrease retinal melatonin levels in rats [9]. Circadian disruption is a key feature of bipolar disorder and severe depression, where alterations in daily rhythms of sleep and activity are most extreme during acute mood episodes, and in bipolar disorder lithium has a stabilising effect on sleep and activity rhythms [8].

As far back as the 1980’s it was hypothesised that people with bipolar disorder may be hypersensitive to light [10, 11], disrupting the entrainment of biological rhythms and leading to the development of mood episodes. Lithium may decrease hypersensitivity via the alteration of retinal physiology [12], as photoreceptors and intrinsically photosensitive retinal ganglion cells (ipRGCs) in the retina convert light to electrical signals which stimulate visual and non-visual responses respectively in the brain. IpRGCs mediate non-visual responses to light via the photopigment melanopsin, and have a direct influence on the circadian clock, which is often disrupted in bipolar disorder. Structural and functional changes in the retina of people with bipolar disorder and depression are well documented [13–17].

The retina is composed of 10 layers of cells which perform a wide range of functions, encompassing both visual and non-visual processing. Its structure and function can be measured using a variety of non-invasive techniques. Optical coherence tomography (OCT) allows direct visualisation and measurement of retinal layers and vasculature. Physiological tests such as electroretinography (ERG), electrooculography (EOG) and dark adaptation thresholds (DATs) [18] can be used to measure specific functions of the retina, allowing the assessment of different types of vision (such as scotopic, mesopic and photopic) or specific retinal cells (such as cones, rods and bipolar cells). Changes in the retina reflect deeper neurodegenerative processes in the brain, and there is increasing interest in using retinal measures to better understand neuropsychiatric disease courses, and as potential biomarkers [19].

Optical coherence tomography (OCT) has found thinning of the retinal nerve fibre later (RNFL) in people with bipolar disorder [13–15]. There is limited evidence about the effect of bipolar disorder on dark adaptation thresholds, but one study has shown increased cone sensitivity in depression [13–15]. Alterations in both cone and rod responses on ERG have been shown in bipolar disorder [17]. A small number of depressed patients (unipolar and bipolar) have been noted to have an abnormally reduced Arden ratio, a measure derived from the EOG which reflects alterations to the retinal pigment epithelium (RPE) with reduced ratios observed in macular dystrophies [16]. The same study found that manic patients had a raised Arden ratio as compared to healthy controls [16]. An important question therefore arises about the effect of lithium on the retina, and whether any changes could contribute to the mediation of disease processes in bipolar disorder and depression.

As outlined above, retinal structure and function can be characterised using several non‑invasive imaging techniques, from which numerous quantitative metrics can be derived. A systematic review of the literature is needed to establish which of these modalities and parameters have previously been evaluated in relation to lithium use and what insights they provide into its potential retinal effects. This systematic review will assess research examining the effect of lithium on any structural or functional outcome of the retina in humans. Due to higher methodological convergence in structural imaging, OCT is the primary outcome and all the functional measures will be treated as secondary.

## Methods

### Search strategy

Systematic searches of the literature were completed using the terms ‘Lithium’ AND ‘retina’ with no restrictions on MEDLINE, Embase, PsychINFO, Commonwealth Agricultural Bureaux (CAB) abstracts, CINAHL and Web of Science from inception until March 2024. The search was repeated in March 2026 to cover the additional two-year period, with the exclusion of CAB abstracts as animal studies had subsequently been excluded. This decision was made due to the heterogeneity and large volume of animal studies, which would have required a more focused strategy to enable a meaningful assessment. More detailed information about the search strategies used can be found in Additional file 1. Forward and backward citation tracking was performed for all papers identified for data extraction. The former was performed using Google Scholar.

### Study inclusion

This review aimed to include all studies measuring the effect of lithium on any outcome reflecting retinal structure or function. Included studies were those employing any design involving a lithium intervention with or without control group comparisons, pre- vs. post-intervention comparisons of lithium, and observational studies where participants are taking lithium. Single case studies, abstracts, expert opinions, duplicates, PhD theses, meeting and conference abstracts, and studies with overlapping samples or with no results were excluded. There were no exclusions based on the quantity or preparation of lithium administered, or the length of administration. Any studies where lithium was administered in combination with another intervention were excluded, unless it was directly compared to the other intervention without lithium.

**Fig. 1:**
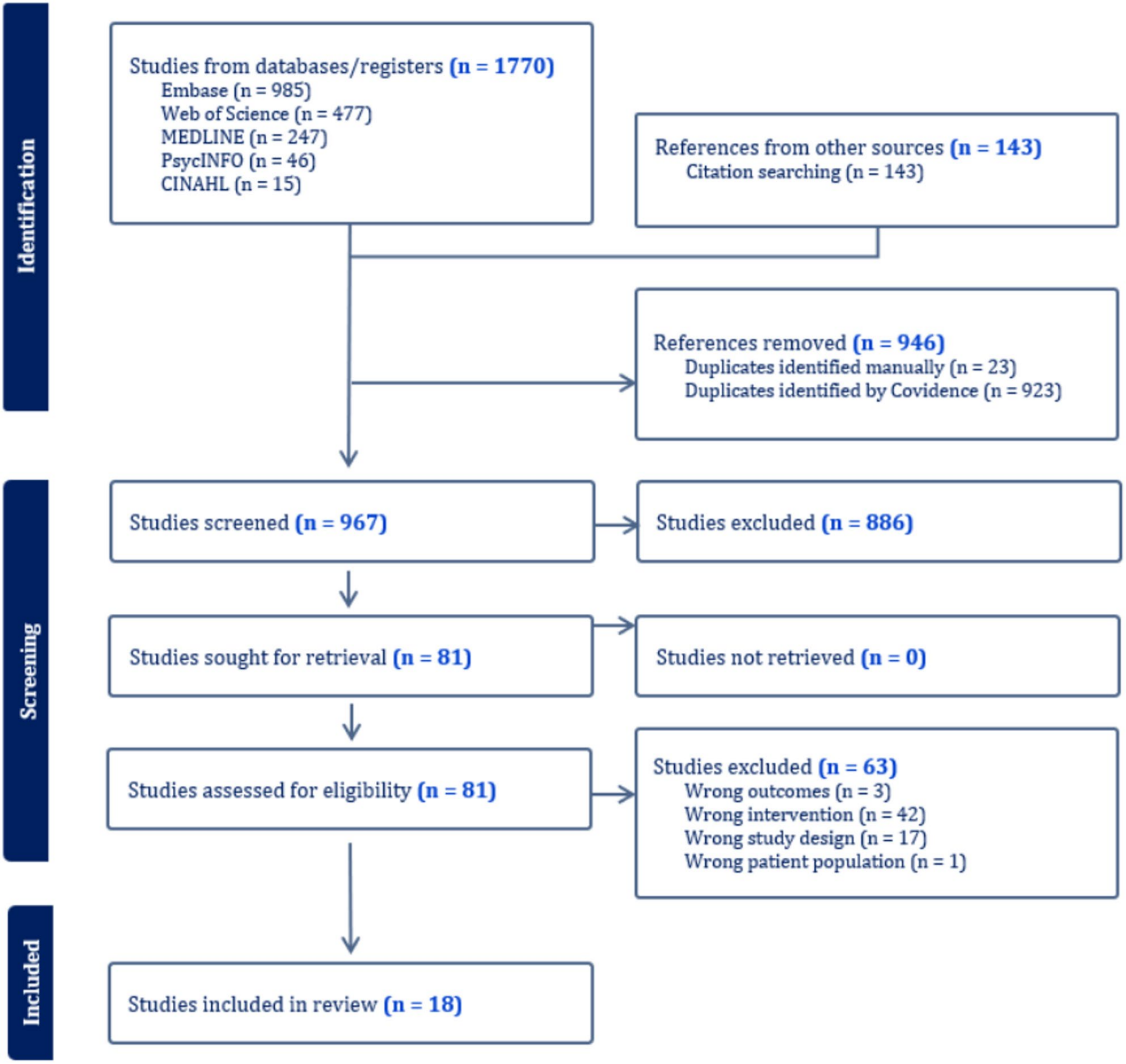
PRISMA Flow diagram outlining the study selection process using covidence

We included studies published in peer-reviewed journals. Non-English language studies were included where possible to reduce language bias, and one paper was translated from German. Title and abstract screening, and subsequent full text review, were conducted independently by two reviewers (NN and DG) using the covidence platform, and any discrepancies resolved through discussion. Figure 1 outlines the process of study exclusion. The study was registered in the PROSPERO database (Number - CRD42024516635).

### Risk of bias assessment

Included studies were assessed using the Joanna Briggs Institute (JBI) critical appraisal tool for Analytical Cross Sectional Studies, the purpose of which is to assess methodological quality and possibility of bias. Reviewers are required to make a yes/no/unclear judgement about whether the following domains are met: clearly defined inclusion criteria; detailed description of study subjects and setting; valid and reliable measurement of exposure; objective standard criteria used for measurement of the condition; identification of confounding factors; strategies to deal with confounding factors; valid and reliable measurement of outcomes; appropriate statistical analysis. If the extracted data was from a subgroup analysis, the JBI criteria were applied to this rather than the overall study methodology. The studies were rated by two independent researchers (NN and DG), and any discrepancies resolved through discussion.

### Outcome measures

The primary outcome of interest were OCT measured structural changes in the thickness of retinal layers. Additional outcomes of interest were any other direct quantitative measure of the structure or function of the retina in response to lithium exposure. Another important imaging technique is OCT‑angiography (OCT-A), which maps blood flow within the retinal and choroidal vascular layers [20]. Measures of retinal function are most commonly derived from electrooculography (EOG), electroretinography (ERG) and dark adaptation thresholds (DATs). DATs measure the absolute intensity threshold for blue light detection over time in scotopic viewing conditions, after the photopigments have been bleached with bright light [20]. The threshold reaches a minimum (highest sensitivity) after around 30 min, with the early component arising from increases in cone sensitivity and the later from increases in rod sensitivity [18]. EOG measures the corneo-fundal potential via electrodes positioned at the lateral and medial canthus of the eye, which decreases in darkness and increases as the eye becomes light adapted [18, 21]. The Arden ratio is derived from the electrooculogram, and is the maximum electrical potential during exposure to light divided by the minimum electrical potential after being in the dark [18, 21]. The EOG can become flat when the RPE-photoreceptor relationship is disrupted [18, 21], and a lower Arden ratio indicates RPE dysfunction [21]. Flash ERG measures the cumulative electrical response from all elements of the retina to a light stimulus, acquired at the surface of the eye [17]. The negative a-wave originates from hyperpolarization of cones and rods, and the positive b-wave from bipolar cells [18]. To assess cones, bright flashes are used in the presence of a background light, for rods lower intensities are used following dark adaptation, and to assess a mixed rod-cone response a bright flash after dark adaptation is used [17]. Only flash ERG was reported in the included studies, and other ERG modalities such as pattern or multifocal ERG were not evaluated. The post illuminatory pupil response (PIPR) quantifies sustained pupil constriction following light offset, typically comparing short- and long-wavelength stimuli as an index of ipRGC-mediated activity.

### Data extraction

Data extraction was conducted independently by two reviewers (NN and DG). Any discrepancies were resolved through discussion. The following data was collected for each included study: Study details (title, author, publication year, journal, doi); Study location (country); Language (If not English); Study design (type of study, duration of study, study setting); Population (healthy controls, conditions); Demographic characteristics including age and sex; Intervention description (lithium formulation, delivery and dosing); Sample size (intervention group, comparison group if applicable); Compliance and adherence assessments; Outcomes measures; Results: all numerical results and any statistics performed.

### Results synthesis

A narrative synthesis and tabulated summary of the included studies was completed due to the diverse range of study designs and heterogenous experimental protocols. Reporting of the systematic review followed PRISMA guidelines. A meta-analysis was not performed given the substantial heterogeneity among included studies, such as differences in the populations taking lithium, control groups, duration and dosing of lithium treatment, outcomes measures, and statistical reporting.

## Results

After completing the search strategy and screening process (Fig. 1), 18 studies were included in the systematic review, and subjected to a risk of bias assessment. The results have been grouped into structural outcomes (*n* = 8) [22–29] and functional outcomes (*n* = 10) [12, 30–38], and summarised in Tables 1 and 2 respectively. For further information about individual studies, including participant demographics, see Additional file 2.

### Risk of bias and quality assessment

**Fig. 2:**
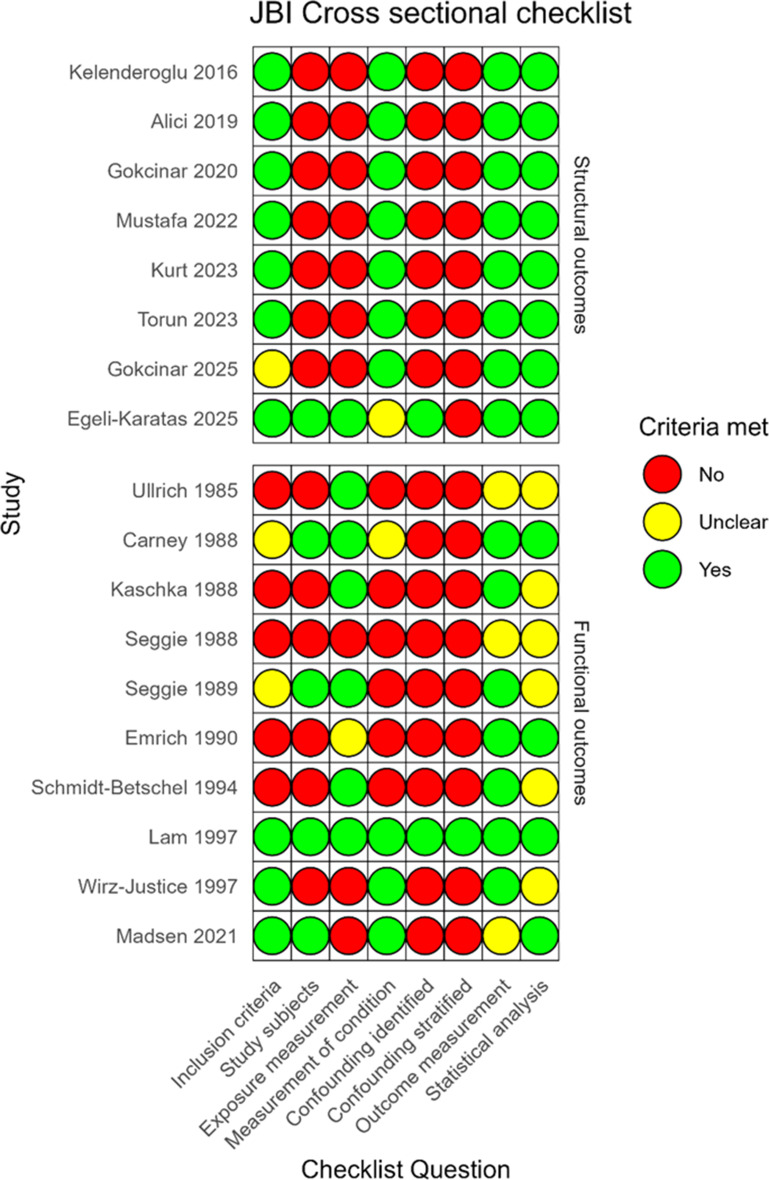
Risk of bias and quality assessment for each included study using the Joanna Briggs Institute Cross-sectional checklist

### Structural measures of the retina

Table 1 outlines the studies measuring structural retinal outcomes, all of which were OCT or OCT-A derived, and of an observational case-control design. There were no studies measuring the effect of lithium on healthy controls - all studies measured outcomes cross-sectionally in participants with bipolar disorder who were already taking lithium to manage their illness. Five of the eight studies compared this group to participants with bipolar disorder not taking lithium, one to healthy controls, and two to both. In six studies, the bipolar disorder participants were euthymic, in one they had variable mood states, and one did not specify. In all but two studies, the comparison was part of a subgroup analysis as opposed to the primary analysis. There were no compliance or adherence measures to confirm therapeutic lithium levels for participants in all but one study, and no information available about formulation, dose, or length of time taken. An additional table [Table 1 in Additional file 3] presents the numerical and statistical information provided by each study.

RNFL was the most commonly reported OCT metric across seven studies. Four studies did not find evidence of a difference in RNFL thickness in participants with bipolar disorder taking lithium. Two compared this group to participants with bipolar disorder not taking lithium [22, 25] but had groups with numbers as low as 3, 5 and 6. Two studies comparing this group to healthy controls had sample sizes of 26 and 36 [26, 27]. An additional paper reported thicker mean RNFL in participants with bipolar disorder taking lithium to those taking antipsychotics and sodium valproate, but although an ANOVA was significant no post hoc analyses were completed to identify the differences more precisely [29].

In studies comparing RNFL in participants with bipolar disorder taking lithium to those taking valproate, two found no evidence of a difference [24, 25], and four found greater thickness in the lithium group [22, 23, 26, 28]. Three studies found no evidence of difference in GCL between participants with bipolar disorder taking lithium and those taking valproate [22, 25, 26], one of which had very small sample size (*n* = 5) [22]. A further study found GCL to be thicker in the lithium group in some regions [23]. The two studies comparing GCL in participants with bipolar disorder taking lithium to those not taking lithium found no evidence of a difference, but had sample sizes as small as 3 and 5 [22, 25]. When compared to healthy controls, the outcomes were conflicting, with one paper suggesting a thinner GCL in the lithium group [27], and the other showing no signs of difference [26].

**Table 1:** Summary of studies measuring the effect of lithium on structural changes in the retina

Paper first author and year	Lithium group description	Non lithium group description	Inclusion criteria	Exclusion criteria	Subgroup analysis	Outcomes	OCT Device	Results summary
Kalenderoglu 2016 [22]	Bipolar disorder taking lithium (*n* = 5)	Bipolar disorder taking valproic acid (*n* = 32) Bipolar disorder not taking lithium or valproic acid (*n* = 6)	Bipolar I disorder (DSM-IV); currently euthymic (confirmed using YMRS and HAM-D); age 18–65; normal ophthalmologic examination	Comorbid first axis psychiatric diagnosis; neurological, immunological or systemic illness; ophthalmologic disease (glaucoma, retinal disease, refraction disturbances)	Yes	Global RNFL GCL	Spectralis, Heidelberg Engineering	RNFL significantly lower in the valproic acid group compared to lithium group There was no evidence of difference in GCL between the three groups
Alici 2019 [23]	Bipolar disorder taking lithium (*n* = 36)	Bipolar disorder taking valproic acid (*n* = 30)	Normal ophthalmologic examination; aged 18–65; euthymic; bipolar disorder type 1	Cooperation problems or cognitive impairment as a result of mental retardation, neurological disease, or alcohol/drug use; electroconvulsive therapy in the last 6 months; history of psychosurgery or other brain surgery, head trauma, alcohol/drug addiction, psychotic symptoms, comorbidity, or any other psychiatric disease; ophthalmologic disease (glaucoma, retinal disease, refraction disturbances)	Yes	**RNFL ** (Global, Inferior Superior) **GCL** (Global, Inferior Superior)	Optovue RTVue device	Significantly increased inferior and global RNFL and GCL in the lithium vs. valproic acid group, no evidence of difference in superior RNFL or GCL.
Gokcinar 2020 [24]	Bipolar disorder taking lithium (*n* = 42)	Bipolar disorder taking valproate (*n* = 28)	Bipolar disorder (in any mood state)	Significant refractive error; BCVA < 20/25 (Snellen); ocular media opacity; glaucoma; retinal disease; history of surgery; presence of chronic systemic diseases such as hypertension and diabetes mellitus	Yes	**Peripapillary RNFL and total retinal thickness** (average, superior, inferior, temporal and nasal) **Macular GCC** (average, superior, inferior, inner superotemporal/ superonasal inferonasal/ inferotemporal, outer superotemporal/ superonasal/ inferonasal) and **total retinal thickness** (outer inferotemporal, central, inner superior/ nasal/ inferior/temporal, outer superior/ nasal/ inferior/ temporal	Nidek RS-3000 Advance Retinascan	No evidence of difference in peripapillary RNFL or macular GCC thickness in all areas
Mustafa 2022 [25]	Bipolar disorder taking lithium monotherapy (*n* = 17)	Bipolar disorder: No Mood stabiliser (*n* = 3) Valproate monotherapy (*n* = 17) Lithium and valproate (*n* = 8)	Bipolar disorder, 6 months remission	Chronic medical diseases such as hypertension, diabetes that may affect eye measurements; neurological diseases that may affect intellectual disability and cognitive functions; smoking, alcohol and psychoactive substance use; in the manic or depressive phase of bipolar disorder; eye disease such as glaucoma, macular abnormalities, high myopia, retinal detachment and ocular trauma.	Yes	**RNFL** (average, superior, nasal, inferior, temporal) **GCIPL** (superior, inferior, average) **RPCP** (superior, nasal, inferior, temporal) **FAZ** VD (superficial, deep)	Nidek RS3000 Advance Retinascan	No evidence of difference in any RNFL, GCIPL, FAZ or RPCP area or deep VD in comparisons of: No MS – Lithium monotherapy; Lithium monotherapy – Valproate monotherapy; Valproate monotherapy – Lithium + Valproate Superficial VD was greater in lithium monotherapy compared to no MS, and lithium + valproate compared to valproate group, but no evidence of difference between lithium monotherapy and valproate monotherapy groups.
Kurt 2023 [26]	Bipolar disorder taking lithium for ≥ 6 months, variable mood states (*n* = 26)	Bipolar disorder taking valproic acid for ≥ 6 months, variable mood states (*n* = 41) Healthy controls (*n* = 49)	Aged 18–65	Intellectual disability, substance abuse, or comorbid psychiatric diseases; neurodegenerative diseases, such as alzheimers disease, parkinsons disease, or multiple sclerosis; glaucoma, retinal laser or surgical intervention histories, or uveitis; spherical values of ± 3.0 diopter and/or a cylindrical value of more than ± 2.0 diopters in auto refractometer measurements; ocular tension values higher than 21 mm/Hg; systematic diseases that affect retinal and choroid thicknesses, such as diabetes mellitus and hypertension; Control group:diagnosed psychiatric diseases; a signal quality of 6 or less in the SD-OCT.	Yes	Right and left: RNFL CMT GCL	Zeis Cirrus 400 model	Significantly thinner left (but not right) RNFL in the valproic acid group as compared to lithium group. No evidence of difference between left or right RFNL in lithium group as compared to controls. No difference in left or right GCL or cmt between the three groups.
Torun 2023 [27]	Bipolar disorder taking lithium (*n* = 39)	Healthy controls (*n* = 36)	Age 18–55; BCVA > 20/25 (Snellen); intraocular pressure under 21 mm Hg; refractive errors fewer than 3 dioptries (spherical equivalent refraction or astigmatism)	History of intraocular surgery, refractive surgery, or trauma, chronic eye conditions (e.g., cataract, strabismus, glaucoma, uveitis, dry eye, age-related macular degeneration, diabetic retinopathy, optic nerve disorders etc.); diabetes mellitus; essential hypertansion; kidney illness; cardiovascular disease; any other neurological disease; using any antipsychotic medication other than lithium	No	Mean RNFL CMT GCL	Spectralis, Heidelberg Engineering	No evidence of difference in RNFL between the two groups. GCL and CMT significantly thinner in bipolar lithium group.
Gokcinar 2025 [28]	Bipolar disorder taking lithium (*n* = 31)	Bipolar disorder taking valproate (*n* = 19)	Bipolar disorder - currently euthymic (HAM-D score < 7 and a YMRS score < 12)	Ocular disease (e.g., glaucoma, macular degeneration); significant refractive error; BCVA < 20/25; history of ocular surgery; chronic systemic conditions (e.g., diabetes mellitus, hypertension); media opacities affecting OCT imaging	Yes	**Peripapillary RNFL** (superior, inferior, nasal, temporal, average (left and right)) **Macular GCC** (superior, inferior, average, left and right eyes)	Nidek RS-3000 Advance Retinascan	Statistically significant difference in inferior quadrant of right and left eyes demonstrating thinner RNFL in the bipolar valproate group as compared to the bipolar lithium group. No statistically significant differences between other RNFL regions or any GCC regions.
Egeli-Karatas [29]	Bipolar disorder type 1 taking lithium (900–1200 mg/day; 0.8–1.1 mEq/L) (*n* = 36)	Bipolar disorder 1 taking valproate (*n* = 36) Bipolar disorder 1 taking antipsychotic (*n* = 28) Healthy controls (*n* = 50)	Age 18–65 Bipolar groups: treated for more than 2 years and using a fixed medication dose for > 3 months; euthymic state for > 6 months	Intellectual disability; substance or alcohol use in the last month; previous brain surgery, or brain trauma; ophthalmological disease; hypertension; diabetes mellitus; inflammatory disease; medication other than mood stabilisers and antipsychotics in the past month Bipolar groups: diagnosis less than 5 years; previous electroconvulsive therapy; comorbid psychiatric disease Healthy controls: psychiatric disease	No	IPL GCL CT RNFL (Nasal, Temporal, Nasal inferior, Nasal superior, Temporal inferior, Temporal superior, global for each measure)	Spectralis, Heidelberg Engineering	Statistically significant difference in mean RNFL, GCL and IPL between the three bipolar lithium groups following an ANOVA, but no post hoc analyses are available to determine which pairwise comparisons are significant, and no comparison to healthy control values.

YMRS, Young Mania Rating Scale; HAM-D, Hamilton Depression Rating Scale; RNFL, retinal nerve fiber layer; GCL, ganglion cell layer; BCVA, Best Corrected Visual Acuity; GCC, Ganglion cell complex; GCIPL, Ganglion cell Inner Plexiform Layer; RPCP, Radial peripapillary capillary plexus; FAZ, foveal avascular zone; VD, vessel density; SD-OCT, Spectral Domain Optical Coherence Tomography; CMT, centrale macular thickness; MS, mood stabiliser; n, number

*All studies were cross-sectional, and no studies completed adherence assessments

### Functional measures of the retina

Table 2 outlines studies measuring one or more functional retinal outcome. Outcomes were measured using a variety of functional tests, including ERG, EOG, DAT and PIPR. Two studies used a single-arm before-after design, five were observational case-control studies, two included both approaches, and a final study used a linear regression model to assess lithium’s contribution in a lager case-control study. Of the single arm before-after studies, two recruited healthy controls, and two recruited people with a specified severe mental illness (SMI) (one psychoses and one depression). Of the observational case-control studies, six compared people with SMI (various specifications) taking lithium to controls, and one compared people with psychoses taking lithium to healthy controls taking lithium. The primary outcome in all but one study was to evaluate the effect of lithium on a functional measure of the retina, and one paper performed a subgroup analysis related to lithium. Seggie et al. [33] describe additional outcomes from the same participants as Carney et al. [31].

Studies examining dark adaptation thresholds were heterogenous, with varied time point measurements and presentation of results. One before-after study noted ‘distinctly impaired adaptometric responses’ in participants with affective or schizoaffective disorders after taking lithium [32], suggesting raised dark adaptation thresholds, but did not specify whether it was the rod or cone mediated phase which was affected. For the cone mediated phase (fast phase), two studies found lower thresholds in participants with bipolar disorder or schizoaffective disorder taking lithium as compared to controls [33, 37], although one only found this difference in male participants [33]. The final study found a higher threshold in one participant with bipolar disorder after taking lithium, comparable to a control [12], indicating reduced cone sensitivity, but their mood state had changed from depressed to euthymic.

One study found complete abolition of the rod mediated phase (slow phase) in 3/10 healthy volunteers taking lithium [30]. Three found higher thresholds in the rod mediated phase in people with SMI taking lithium (as compared to controls) [31, 34, 37], and a further study found higher thresholds in male patients as compared to both female patients and male controls during the rod mediated phase [33]. One noted a significant increase in DAT [34] in healthy controls taking lithium at 20 min, as compared to healthy controls not taking lithium.

In the two before and after studies reporting the Arden ratio after lithium administration, one found a statistically significant mean decrease on day 5 and 10 in healthy controls [30], while the other found lower mean values in people with psychosis, but did not report whether these were statistically significant [32]. This was not the case for all eyes or all participants. In two comparisons of people with mental illness taking lithium to controls, no difference was seen in the Arden ratio [36, 37], including when correlated to the length of lithium use [37]. One further study found a decreased Arden ratio [35] in people taking lithium as compared to controls, irrespective of length of lithium use. The participant groups were small and statistical information was limited.

In respect to ERG, the only included before and after study of lithium use in healthy controls [30] found no evidence of alterations in a- or b-wave morphology, and therefore no clear effect on bipolar cells or photoreceptors. The two studies comparing ERG outcomes in people with mental illness taking lithium to controls found no evidence of a difference in b-wave amplitude or latency [36, 37].

**Table 2:** Summary of studies measuring functional changes in the retina following lithium use

Paper first author and year	Study design	Lithium group description	Non lithium group description	Inclusion criteria	Exclusion criteria	Adherence assessment	Subgroup analysis	Outcomes	Summary
Ullrich 1985 [30]	Single arm before- after	Healthy volunteers before taking lithium and after taking lithium for 10–21 days (*n* = 10)		Aged 20–30 Healthy	Eye abnormalities	Lithium level 0.6–0.8	No	DAT	Complete abolition of the slow phase of dark-adaptation in three participants taking lithium, which was reversed on cessation. The adaptation properties in photopic and mesopic conditions were constant during and after lithium administration.
								EOG	Taking an average of both eyes, there was a decrease (*p*<.05, one-tailed) in the mean Arden-ratio of 5% on day 5 and 10% on day 10 of lithium treatment compared to pre-treatment values. Fourteen days after lithium discontinuation the mean Arden-ratio increased again to above the pretreatment value (*p*<.05) value. Changes were not related to serum lithium levels or time taking lithium. Four participants showed a ‘significant’ decrease in the Arden-ratio in both eyes during lithium treatment, three a decrease in only one eye (relatively constant in the other), one no change in either eye, and one an increase in both eyes.
								ERG	No clear effect of the lithium regime was found on inter or intra-individual a-wave or b-wave amplitudes or latencies. A non-significant tendency towards a shortening of photopic ERG latencies of a- and to a smaller degree b-waves was demonstrated.
Carney et al. 1988 [31]	Observational case-control	History of mania or hypomania, taking lithium, euthymic (*n* = 19)	Age and sex matched controls taking no medication (*n* = 19)	As per group description	Patient group: other psychiatric or medical illness; uncorrected visual disorders	Lithium levels 0.60–0.98 mmol/l	No	DAT	Significant increase in dark adaptation threshold at 10, 15, 20, 25 and 30 min time points, and the finishing threshold, in the lithium group as compared to controls. Differences between the groups were twofold at nearly all time points, and increased with the length of time in darkness. A higher threshold was found for male patients and male controls in the first 9 min. Male patients had a higher threshold than male controls and female patients at minutes 10, 15, 25, and 30. Patients with a family history of psychiatric illness other than affective disorder had lower descending thresholds and finishing thresholds. A positive correlation was found between the total number of episodes of depression, mania and hypomania, and the first descending threshold.
Kaschka et al. 1988 [32]	Single arm before- after	Affective and schizoaffective psychoses, prior to commencing lithium and after lithium initiation during the study (unknown duration) (*n* = 19)		Affective and schizoaffective psychoses	None specified	Serum lithium level (not further defined)	No	DAT	‘Distinctly impaired adaptometric responses’ observed in 6 of 16 participants when taking lithium at a therapeutic level. No relationship was found between dark adaptation and DSM-III diagnoses or Hamilton scores.
								EOG	In 10 participants there was a statistically significant decrease in Arden ratio (6 repeats) in one or both eyes after lithium administration, and there was no difference in either eye in the remaining 9 participants. The mean Arden ratio for right eyes, left eyes, and both eyes was lower post lithium administration. No relationship between electooculographic results and DSM-III diagnoses or Hamilton score.
Seggie 1988 [12]	Observational case-control Single arm before-after	Depressive or bipolar disorder after lithium administration for 1–3 weeks (*n* = 1)	Depressive or bipolar disorder before lithium administration (*n* = 1) Healthy controls (*n* = 1)	None specified	None specified	None	No	DAT	During the first five minutes, the depressed participant was able to detect light intensity ten times smaller than that detected by the control. Following initiation of lithium, and whilst euthymic, the participants sensitivity to light was normalised in the first 5 min of DAT to control values. For the 6–10 min response period the minimal detected light intensity was less than the control participant. After 15 min, light adaptation was the same for the control and depressed participant both whilst unmedicated and taking lithium.
Seggie 1989 [33]	Observational case-control	History of mania or hypomania, taking lithium, euthymic (*n* = 19)	Age and sex matched controls taking no medication (*n* = 17)	As per group description	Patient group: other psychiatric or medical illness; uncorrected visual disorders	Mean serum lithium: 0.72mmol/L Range: 0.60–0.98	No	DAT	Control subjects demonstrated the expected decrease in DAT over time for both the cone and rod responses. Bipolar lithium participants also demonstrated a significant fall in DAT over time for both the cone and rod responses, but for the rod portion male patients had a higher threshold than female patients. Male controls had statistically significant lower cone thresholds than male bipolar participants with slower adaptation, and lower rod thresholds. No significant differences in threshold between female patients and female controls.
Emrich et al. 1990 [34]	Observational case- control Single arm before-after	Affective or schizoaffective psychosis on a continuous lithium regime during a symptom free period (*n* = 30) Healthy volunteers after 10–19 days oral lithium (*n* = 15)	Healthy volunteers before lithium exposure (*n* = 15)	None specified	None specified	Lithium level 0.4–1.1 meq/liter for psychosis group, 0.4–0.8 for healthy control group	No	DAT	Significant decrease in scotopic sensitivity (increase in dark adaptation) in healthy volunteers following lithium exposure, returned to normal on discontinuation. No significant difference between dark adaptation values in the participants with psychoses on continuous lithium and healthy controls taking lithium. Three of 30 participants in lithium group had normal dark adaptation, one of whom was a poor lithium responder, and two of whom this information was not available due to later discontinuation of lithium. The remaining 27 displayed increased dark adaptation thresholds, 26 were partial or complete lithium responders. A subgroup analysis on 7 participants with affective psychoses before and after lithium administration found a decrease in scotopic sensitivity following lithium.
Schmidt-Betschel 1994 [35]	Observational case-control	Patients treated with lithium carbonate (*n* = 10)	No lithium therapy (*n* = not specified)	None specified	None specified	Lithium level ‘therapeutic range’ Mean 0.80 ± SD 0.14 mmol/l	No	EOG	Baseline potential (after 30 min of dark adaptation) elevated in the lithium group as compared to the control group. After the illumination was increased there was no statistically significant difference in potential. The Arden ratio was significantly reduced in the lithium group as compared to controls. The amplitude of the baseline potentials and potentials under illumination, and the Arden ratio, were independent of the length of lithium use.
Lam 1997 [36]	Observational case-control	Chronic lithium use for 3 years or more, currently euthymic (*n* = 24)	Healthy controls (*n* = 21)	Age 18–65 years Lithium Group: regular lithium for at least 2 years; euthymic mood (less than 8 on the (HDRS), and less than 6 on the (YMRS) Control group: No history or family history of mood disorder; drug-free	History of ocular or retinal disease prior to lithium use; history of chronic (> 6 months) or current use of other medications with known retinal effects; ophthalmological abnormalities	Lithium level range 0.5–1.2 mEq/L. Mean ± SD: 0.8 ± 0.2	No	EOG	No significant differences were found between the groups (or by eye) for EOG ratios, or dark-adapted and light-adapted EOG amplitudes.
								ERG	No significant between group differences were found between b-wave amplitude and latency times.
Wirz-Justice 1997 [37]	Observational case- control	Bipolar disorder or schizoaffective disorder - taking lithium (*n* = 71)	Age matched controls (*n* = 33) from database	No complaints of visual problems or history of eye disease	Not specified	None	No	DAT	The lithium group demonstrated a lower cone adaptation threshold at 5 min than the control group, and higher rod adaptation thresholds. No significant correlation with duration of lithium treatment.
								EOG	No marked abnormalities in EOG measurements including the Arden ratio when comparing groups, and no correlation in these measures with the length of lithium treatment.
								ERG	No abnormalities in scotopic or photopic ERGs (b wave latency and amplitude). Single eyes had longer latencies or lower amplitudes but this was not consistent over all conditions or correlated with duration of lithium treatment.
Madsen 2021 [38]	Cross-sectional linear regression model	Recent bipolar diagnosis (any mood state) taking lithium ( *n* = 12)	Recent bipolar diagnosis (any mood state) not taking lithium (*n* = 19)	Recent bipolar disorder diagnosis	Prior or current eye disorder, eye trauma, eye surgery; family history of glaucoma	None	Yes	PIPR	No significant association between PIPR and lithium use.

EOG, electrooculogram; ERG, electroretinogram; n, number; PIPR, post illumination pupillary response; HDRS, Hamilton Depression Rating Scale; YMRS, Young Mania Rating Scale

## Discussion

### Main findings

The effect of lithium on retinal structure and function was systematically reviewed, finding large heterogeneity between studies’ samples and methods, with many limitations which make it hard to produce solid generalisations. No clear conclusions can be drawn for the effect of lithium on total retinal thickness, GCC, CMT, RPCP, FAZ or VD because of the paucity of high quality data. More studies are needed to identify if there is an effect of lithium on these measures.

RNFL was reported in 7 of the 8 structural papers and as such some tentative inferences can be made. The two studies comparing participants with bipolar disorder not taking lithium to healthy controls [26, 27] found no difference in RNFL thickness between the groups. Given that people with bipolar disorder have a thinner RNFL than people without bipolar disorder across most regions [13–15], these results could indicate that lithium is protective against such thinning. In studies comparing RNFL in participants with bipolar disorder taking lithium to those taking valproate, two found no evidence of a difference [24, 25], and four found greater thickness in the lithium group [22, 23, 26, 28]. As above, this could suggest a protective effect of lithium, but to definitively answer this question requires an appropriately designed and powered study to compare participant groups.

One study included participants with bipolar disorder in various mood states [24], which has been previously linked to heterogeneity in regions of altered thickness, and which was not accounted for as a covariate [14]. Overall, there is a suggestion that lithium may protect against thinning of the RNFL in people with bipolar disorder, but the evidence is not strong.

The evidence base for GCL it is difficult to assess as there was no clear trend in the included studies. In previous research, no significant difference in GCL between people with bipolar disorder and healthy controls has been suggested [13, 15], although there has been a trend towards this being thinner in people with bipolar disorder. We did not identify any research examining the effect of lithium on RNFL or GCL in healthy subjects, which limits conclusions about its effect on the retina in the general population.

Only one study examined baseline EOG and PIPR and so no trends for these two measures can be identified. No association was found between PIPR (an index of ipRGC function) and lithium use in study participants with bipolar disorder [38], although the sample size was small, and there was a reliance on a single trial per participant.

For the dark adaptation threshold cone mediated phase, results could indicate increased cone sensitivity in response to lithium, but mental illness is a possible confounder. There is limited evidence on the effect of bipolar disorder or depression on dark adaptation, but one previous study found increased cone sensitivity in bipolar disorder [39]. Given the much larger sample sizes of the first two studies [33, 37], evidence suggests an increase in cone sensitivity in people with mental illness taking lithium as compared to controls. Despite limitations, the results from included studies indicate decreased rod sensitivity (a scotopic deficit) as an effect of lithium, although mental illness may again have been a confounding factor in some.

Overall, results from included studies suggest that the Arden ratio could be decreased by lithium, which corresponds to RPE dysfunction, but larger and more robust studies are required to answer this question definitively. As the Arden ratio was not reduced in all eyes or all participants, this suggests any effect of lithium on the Arden ratio may only be seen in a subgroup of people. Mental illness was a possible confounding factor in some studies, although no established evidence could be found as to the effect of mental illness on the Arden ratio.

No difference was found in ERG changes between people with bipolar disorder taking lithium and healthy controls in the included studies. Given that prolonged b-wave latency (cone response) and decreased b-wave amplitude (rod and mixed rod-cone responses) have been shown in people with bipolar disorder as compared to controls [17], these studies could indicate a reversal of these effects from lithium. However, other research has found no difference in ERG measures in participants with bipolar disorder as compared to controls [40], which would point towards no effect of lithium in the above studies. There is no evidence to suggest lithium has an effect on ERG, but the study designs limit inference.

### Limitations

There were multiple limitations in studies measuring retinal structure. Most studies performed subgroup analyses (6/8), and were therefore not designed to answer questions about the effect of lithium on the retina, as acknowledged by some authors [22]. The risk of bias assessment (Fig. 2) highlighted the paucity of participant demographic and exposure measurement information, as well as lack of recognition and accounting for confounding in the majority of studies. Possible differences in age, gender, and clinical variables increase the risk of potential confounding accounting for any differences. In particular, it is known that increasing age correlates with decreased thickness of multiple retinal layers [41]. The consistent lack of information about the exposure across studies (i.e. lithium dose, level and duration) is another significant limitation, as lithium response time is highly variable [42], and the dose and length of lithium administration may have cumulative effects on any retinal changes observed. The number of participants in each group were often small and unevenly distributed due to the subsidiary nature of the analyses, reducing validity of the results. Five different OCT scanners were used across these studies, which makes direct comparison of the results less reliable. There were no studies measuring the effect of lithium in healthy controls, and so the effect on this group is not known. All study participants taking lithium had a diagnosis of bipolar disorder, and there was variation in the characteristics of the comparison groups (bipolar disorder taking valproate; bipolar disorder not taking lithium; bipolar disorder taking antipsychotic; healthy controls). As such, limited inference can be made from the results but general trends are of interest and may help to direct future research.

All studies examining functional outcomes of the retina had significant limitations. All but one failed to identify or account for confounding factors, and half did not adequately describe participants (Fig. 2). Furthermore, the assessment of bipolar disorder was inadequate in the majority of studies (Fig. 2). Not all participants with bipolar disorder were euthymic at the time of testing, which may have impacted the results. The inclusion criteria were not clearly stated in the majority of studies (Fig. 2), introducing potential bias to these studies.

### Scientific and clinical implications

Given the multiple and varied limitations of the included studies, it is difficult to infer any definitive differences in OCT measures in people taking lithium. Despite this, it seems possible that lithium does have an impact on the structure of the retina, but that further evidence is required to answer this question. Although difficult to conduct due to ethical constraints, randomised control trials assessing the effect of lithium on OCT measures in healthy controls would be desirable to gain a better understanding of how it may affect the retina. Alternatively, larger cross-sectional studies with purposeful samples of participants with bipolar disorder or depression taking lithium and not taking lithium, and healthy controls, that are appropriately powered, would be desirable. This would have fewer ethical considerations as lithium would not need to be initiated, although variable length of treatment would need to be accounted for. To improve validity of future results, consistency of OCT scanners should be coordinated, and the regions of the retina to be analysed should be standardised. In addition, studies should measure serum lithium levels to ensure they are within the therapeutic range, and the length of lithium use should be recorded. Given that effects of lithium may increase over time, longitudinal follow-up should be considered.

If lithium is associated with structural changes in the retina, particularly in protecting against RNFL changes in individuals with bipolar disorder and/or depression, this could have implications for both clinical practice and scientific research. The retina is an extension of the central nervous system with which it shares its embryonic origin, and as such is considered an accessible window into the brain [43]. Lithium induced changes to this unique structure may therefore provide valuable insights into its effects on other brain regions, as well as potentially providing an objective predictive measure of treatment response. Furthermore, if lithium causes structural or functional changes to the retina, researchers could explore potential correlations between lithium induced retinal changes, and downstream non-visual effects of light in the brain, including circadian phenotyping of melatonin measurements.

Despite the incomplete evidence base, this review allows consideration of the effect of lithium on the structure and function of the retina, how these may relate to one another, and where important data is missing to evaluate this. For example, it is suggested that baseline EOG may be increased following lithium administration, but corresponding differences in the RPE on OCT have not been measured. Functional measures may be more sensitive to initial changes in the retina, and it will therefore be important to expand the evidence base in this area. An attempt to correlate these to structural changes, which are more likely to be identified after prolonged lithium administration, is needed, and a multimodal approach desirable to fully understand the implications of any observed changes.

## Conclusion

Studies examining the effect of lithium on the *structure* of the retina used up to date OCT techniques but were mostly subgroup analyses with small numbers of undefined participants, and with no accounting for confounding factors. There was no information about the effect of lithium in healthy controls, but some suggestion that it may be protective against thinning of the RNFL in people with bipolar disorder. Studies examining the effect of lithium on the *function* of the retina were older with a similar lack of accounting for confounding factors. There was some limited data to support that people with bipolar disorder taking lithium had increased cone and decreased rod sensitivity as compared to controls. Due to the poor quality of the evidence only limited conclusions can be made, and larger more robust studies are needed.

## Supplementary Information

Below is the link to the electronic supplementary material.

Supplementary Material 1

Supplementary Material 2

Supplementary Material 3

## Data Availability

All data generated during this study are included in this published article [and its supplementary information files].

## Abbreviations

RNFL: Retinal nerve fibre layer
JBI: Joanna Briggs Institute
OCT: Optical coherence tomography
ERG: Electroretinography
EOG: Electrooculography
DAT: Dark adaptation thresholds
ipRGC: intrinsically sensitive retinal ganglion cells
RPE: Retinal pigment epithelium
CAB: Commonwealth Agricultural Bureaux
OCT-A: OCT-angiography
PIPR: Post-illuminatory pupillary response
YMRS: Young Mania Rating Scale
HAM-D: Hamilton Depression Rating Scale
GCL: Ganglion cell layer
BCVA: Best Corrected Visual Acuity
GCC: Ganglion cell complex
GCIPL: Ganglion cell Inner Plexiform Layer
RPCP: Radial peripapillary capillary plexus
FAZ: Foveal avascular zone; VD, vessel density
SD-OCT: Spectral Domain Optical Coherence Tomography
CMT: Centrale macular thickness
MS: Mood stabiliser
N: Number
