# Supplementary material for: The effect of lithium on the structure and function of the human retina: a systematic review
Source: BMC Ophthalmol. 2026 Jul 29;26:448. doi: 10.1186/s12886-026-05095-y (PMC13422349; doi:10.1186/s12886-026-05095-y)
Supplement: Supplementary file 1 — Supplementary Material 1 [file 12886_2026_5095_MOESM1_ESM.docx]

Systematic Review Search history

**Embase**

Embase Classic+Embase <1947 to 2024 February 12>

1 exp lithium/ 66346

2 exp lithium fluoride/ or exp aluminum lithium hydride/ or exp lithium blood level/ or exp lithium therapeutic drug test kit/ or exp lithium acetate/ or lithium gluconate/ or exp lithium hydroxybutyrate/ or exp lithium salt/ or exp lithium intoxication/ or exp lithium carbonate/ or exp lithium derivative/ or exp lithium nephropathy/ or exp succinate lithium/ or exp lithium sulfate/ or exp lithium citrate/ or exp lithium chloride/ or exp lithium electrode/ or exp lithium ion/ 39339

3 lithium.tw. 68835

4 1 or 2 or 3 114596

5 exp retina/ 182331

6 exp retina macula hole/ or exp retina macula lutea/ or exp retina dystrophy/ or exp "RMC cell line (retina)"/ or exp peripheral retina/ or exp retina hemorrhage/ or exp retina detachment/ or exp retina cone/ or exp retina vein occlusion/ or exp retina macula translocation/ or exp retina horizontal nerve cell/ or exp retina pigment epitheliopathy/ or exp retina vasculitis/ or exp retina disease/ or exp retina angiography/ or exp retina macula cystoid edema/ or exp retina malformation/ or exp retina bipolar ganglion cell/ or exp retina blood vessel occlusion/ or exp "rMC-1 cell line (retina)"/ or exp retina injury/ or exp retina examination/ or exp retina pigment cell/ or exp retina antigen/ or exp retina receptive field/ or exp retina S antigen/ or exp retina blood flow/ or exp retina cell/ or exp retina fluorescein angiography/ or exp retina tumor/ or exp retina pigment degeneration/ or exp nasal retina/ or exp retina Y cell/ or exp retina development/ or exp retina vein/ or exp retina macula hemorrhage/ or exp retina detachment balloon catheter/ or exp retina X cell/ or exp retina degeneration/ or exp central retina artery occlusion/ or exp central retina/ or exp retina ischemia/ or exp retina gyrate atrophy/ or exp retina image/ or exp retina rod/ or exp retina capillary/ or exp retina surgery/ or exp retina detachment surgery/ or exp retina ganglion cell/ or exp retina fold/ or exp retina tear/ or exp retina edema/ or exp "retina and media testing equipment"/ or exp blood retina barrier/ or exp retina fovea/ or exp retina blood vessel/ or exp isolated retina/ or exp retina necrosis/ or exp central retina vein occlusion/ or exp retina exudate/ or exp retina amacrine cell/ or exp retina arteritis/ or exp retina nerve cell/ or exp "HRMEC cell line (retina)"/ or exp retina maculopathy/ or exp retina neovascularization/ or exp retina tissue/ or exp inner retina/ 456382

7 retina*.tw. 304053

8 5 or 6 or 7 568338

9 4 and 8 471

Rerun on 6^th^ March 2026 to cover the years 2024 to 2026. Results = 44. (Search strategy not saved but was repeated from above)

**APA PsychInfo**

APA PsycInfo <1806 to February Week 2 2024>

1 exp Lithium Carbonate/ or exp Lithium/ 6966

2 lithium.tw. 11855

3 exp Retina/ or exp "Ganglion Cells (Retina)"/ 9196

4 retina*.tw. 19870

5 1 or 2 11938

6 3 or 4 21253

7 5 and 6 22

Rerun on 6^th^ March 2026 to cover the years 2024 to 2026. Results = 2. (Search strategy not saved but was repeated from above)

**Medline**

Ovid MEDLINE(R) and Epub Ahead of Print, In-Process, In-Data-Review & Other Non-Indexed Citations, Daily and Versions <1946 to February 12, 2024>

1 exp Lithium/ 23791

2 exp Lithium Chloride/ 3897

3 exp Lithium Compounds/ 10103

4 exp Lithium Carbonate/ 2982

5 lithium.tw. 63475

6 2 or 3 or 4 or 5 66591

7 exp Retina/ 155081

8 retina*.tw. 226125

9 7 or 8 276694

10 6 and 9 138

Rerun on 6^th^ March 2026 to cover the years 2024 to 2026. Results = 109. (Search strategy not saved but was repeated from above)

**Web of science**

lithium (Topic) and retina* (Topic) and Preprint Citation Index (Exclude – Database)

4th March 2024

Results: 402

lithium (Topic) and retina* (Topic) and Preprint Citation Index (Exclude – Database) and 2026 or 2025 or 2024 (Publication Years) and Patent (Exclude – Document Types)

6th March 2026

Results: 25

**CINHL**

Search below completed on 23/02/2024. Results: 15. Rerun on 06/03/26 with no additional results.

| S8 | S4 AND S7 | Expanders - Apply equivalent subjects Search modes - Find all my search terms | Interface - EBSCOhost Research Databases Search Screen - Advanced Search Database - CINAHL Plus | 15 |
| --- | --- | --- | --- | --- |
| S7 | S5 OR S6 | Expanders - Apply equivalent subjects Search modes - Find all my search terms | Interface - EBSCOhost Research Databases Search Screen - Advanced Search Database - CINAHL Plus | 39,015 |
| S6 | AB retina* OR TI retina* | Expanders - Apply equivalent subjects Search modes - Find all my search terms | Interface - EBSCOhost Research Databases Search Screen - Advanced Search Database - CINAHL Plus | 20,914 |
| S5 | (MH "Retina+") OR (MH "Retinal Ganglion Cells") OR (MH "Retinal Detachment") OR (MH "Retinal Diseases+") OR (MH "Cone-Rod Dystrophies") OR (MH "White Dot Syndromes") OR (MH "Cone Dystrophy") OR (MH "Synucleins") OR (MH "Coloboma+") OR (MH "Kearns-Sayre Syndrome") | Expanders - Apply equivalent subjects Search modes - Find all my search terms | Interface - EBSCOhost Research Databases Search Screen - Advanced Search Database - CINAHL Plus | 30,445 |
| S4 | S2 OR S3 | Expanders - Apply equivalent subjects Search modes - Find all my search terms | Interface - EBSCOhost Research Databases Search Screen - Advanced Search Database - CINAHL Plus | 4,980 |
| S3 | AB lithium OR TI lithium | Expanders - Apply equivalent subjects Search modes - Find all my search terms | Interface - EBSCOhost Research Databases Search Screen - Advanced Search Database - CINAHL Plus | 4,008 |
| S2 | (MH "Lithium") OR (MH "Lithium Compounds+") OR (MH "Lithium Carbonate") | Expanders - Apply equivalent subjects Search modes - Find all my search terms | Interface - EBSCOhost Research Databases Search Screen - Advanced Search Database - CINAHL Plus | 3,073 |
| S1 | (MH "Lithium") | Expanders - Apply equivalent subjects Search modes - Find all my search terms | Interface - EBSCOhost Research Databases Search Screen - Advanced Search Database - CINAHL Plus | 2,135 |
