# Supplementary material for: The effect of lithium on the structure and function of the human retina: a systematic review
Source: BMC Ophthalmol. 2026 Jul 29;26:448. doi: 10.1186/s12886-026-05095-y (PMC13422349; doi:10.1186/s12886-026-05095-y)
Supplement: Supplementary file 2 — Supplementary Material 2 [file 12886_2026_5095_MOESM2_ESM.docx]

Table 1: Additional information for individual studies measuring structural outcomes of the retina

| **Paper first author and year** | **Journal** | **Country** | **Demographics lithium group** | **Demographics comparison group** |
| --- | --- | --- | --- | --- |
| Kalenderoglu 2016 (22) | Comprehensive Psychiatry | Turkey | Not specified (subgroup analysis) | Not specified (subgroup analysis) |
| Alici 2019 (23) | Arch Clin Psychiatry | Turkey | Not specified (subgroup analysis) | Not specified (subgroup analysis) |
| Gokcinar 2020 (24) | Asia Pac Psychiatry | Turkey | Not specified (subgroup analysis) | Not specified (subgroup analysis) |
| Mustafa 2022 (25) | Journal of Affective Disorders | Turkey | Not specified (subgroup analysis) | Not specified (subgroup analysis) |
| Kurt 2023 (26) | Psychiatry and Clinical Psychopharmacology | Turkey | Not specified (subgroup analysis) | Healthy controls:  Age 42.06 ± 12.10 (mean ± SD); Male = 36.7%  Bipolar disorder – valproic acid: Not specified (subgroup analysis)  Bipolar disorder lithium and bipolar disoder valproic acid combined: Age 39.78 ± 11.78 (mean ± SD); Male = 49.3% |
| Torun 2023 (27) | Photodiagnosis and photodynamic therapy | Turkey | Age: 36.15±9 (mean ± SD); Male = 10 (25.6%) | Age: 33.33±7.8 (mean ± SD); Male = 8 (22.2%) |
| Gokcinar 2025 (28) | Psychiatry Research | Turkey | Not specified (subgroup analysis) | Not specified (subgroup analysis) |
| Egeli-Karatas (29) | Psychiatria Danubina | Turkey | Age = 39.72 +-1.92 (mean ± SD); Female = 19 (52.8%); Smoking: yes = 15 (41.7%); no = 21 (58.3%) | Healthy controls:  Age 38.17 +-1.98 (mean ± SD); Female = 32(64%), Smoking = 8 (16%)    Bipolar disorder taking valproate:  Age 40.88+-1.61 (mean ± SD), female = 19(52.8%), smoking = 15 (41.7%)    Bipolar disorder taking antipsychotic  Age = 38.92+-1.74 (mean ± SD), female = 10 (35.7%), smoking = 11 (39.3%) |

Table 2: Additional information for individual studies measuring functional outcomes of the retina

| **Paper first author and year** | **Journal** | **Country** | **Demographics lithium group** | **Demographics comparison group** |
| --- | --- | --- | --- | --- |
| Ullrich 1985 (32) | Acta psychiatr. scand | Germany | Not specified | NA |
| Carney et al 1988 (31) | Pharmacopsychiat | Canada | Age = 53 (mean); 25-67 (range) ; Male = 12, Female = 7 | Age = 45 (mean); 21-66 (range); Male = 12, Female= 7 |
| Kaschka et al 1988 (33) | Pharmacopsychiat | Germany | Age: 21-64 (range); 39.8 (median); Male = 5, Female = 14 | Age: 21-64 (range); 39.8 (median); Male = 5, Female = 14 |
| Seggie 1988 (14) | Prog Neuro-psychopharmacol & Biol Psychiat | Cananda | Age - 33, Female | Age -24, Female |
| Seggie 1989 (30) | Prog Neuro-psychopharmacol & Biol Psychiat | Canada | Age = 53 (mean); 25-67 (range); Male = 12, Female = 7 | Age = 45 (mean); 21-66 (range); Male = 12, Female= 7 |
| Emrich et al 1990 (34) | Am J Psychiatry | Germany | Not specified | Not specified |
| Schmidt-Betschel 1994 (35) | Der Ophthalmologe | Germany | Age = 52,7 ± 13,1 (mean ± SD); 36-73 (range) | Not specified |
| Lam 1997 (36) | Biol Psychiatry | Canada | Age 48.9 +- 11.5 (mean ± SD); 24-65 (range); Male = 7, Female = 17 | Age 45.7 -+  7.4 (mean ± SD); 30-63 (range); Male = 7, Female = 14 |
| Wirz-Justice 1997 (37) | Biol Psychiatry | Switzerland | Age - 54.8 (mean), 23-80 (range); Male = 27, Female = 44 | ‘Age matched’, not further specified |
| Madsen 2021 (38) | Int J Bipolar Disord | Denmark | Not specified (subgroup analysis) | Not specified (subgroup analysis) |
