# Supplementary material for: The effect of lithium on the structure and function of the human retina: a systematic review
Source: BMC Ophthalmol. 2026 Jul 29;26:448. doi: 10.1186/s12886-026-05095-y (PMC13422349; doi:10.1186/s12886-026-05095-y)
Supplement: Supplementary file 3 — Supplementary Material 3 [file 12886_2026_5095_MOESM3_ESM.docx]

Table 1: Summary of data and statistics for individual studies measuring structural outcomes of the retina

| Paper |  | Results | | | | | | | | |  | |
| --- | --- | --- | --- | --- | --- | --- | --- | --- | --- | --- | --- | --- |
| Kalenderoglu 2016 | **OCT measure** | **Bipolar lithium (n = 5)** | | | **Bipolar valproate (n = 32)** | | **Bipolar no mood stabiliser (n=6)** | |  | | |  |
|  | GCL values | No evidence of a statistically significant difference between groups | | | | | | |  | | |  |
|  | RNFL | RNFL significantly lower in patients using valproic acid compared to the other 2 groups (p = 0.004) | | | | | | |  | | |  |
| Alici 2019 | **OCT Measure** | **Lithium (n = 36)** **mean ± sd** | | | **Valproic acid (n = 30)** **mean ± sd** | | **Statistic** | | **P value** | | |  |
|  | RNFLT superior | 109.3553 ± 11.0719 μm | | | 105.4829 ± 16.2090 μm | | Mann Whitney test | | 0.209 | | |  |
|  | RNFLT inferior | 107.0535 ± 9.5737 μm | | | 101.2387 ± 10.2108 μm | | Mann Whitney test | | 0.016 | | |  |
|  | RNFLT global | 108.1990 ± 9.0703 μm | | | 103.3808 ± 11.6999 μm | | Mann Whitney test | | 0.028 | | |  |
|  | GCL Superior | 95.6779 ± 5.3231 mm³ | | | 93.2546 ± 8.5765 mm³ | | Mann Whitney test | | 0.073 | | |  |
|  | GCL Inferior | 96.9529 ± 4.2096 mm³ | | | 93.1591 ± 8.4444 mm³ | | Mann Whitney test | | 0.028 | | |  |
|  | GCL global | 96.3100 ± 4.6009 mm³ | | | 93.1128 ± 8.5045 mm³ | | Mann Whitney test | | 0.041 | | |  |
| Gokcinar 2020 | **OCT Measure** | | | | | | **Bipolar lithium (n = 42)** | | **Bipolar valproate (n = 28)** | | |  |
|  | Peripapillary RNFL and total retinal thickness (average, superior, inferior, temporal and nasal)  Macular GCC (average, superior, inferior, inner superotemporal/ superonasal inferonasal/ inferotemporal, outer superotemporal/ superonasal/ inferonasal) and total retinal thickness (outer inferotemporal, central, inner superior/ nasal/ inferior/temporal, outer superior/ nasal/ inferior/ temporal | | | | | | p>0.05 for all measures | | | |  | |
| Mustafa 2022 | **OCT outcome** | **Bipolar no mood stabiliser (n = 3)** | | | **Bipolar lithium monotherapy (n=17)** | | **Bipolar valproate (n = 17)** | | **Bipolar – lithium and valproate (n = 8)** | | | **p-value (Kruskall Wallis)** |
|  | RNFL average | 113 ± 5.291 | | | 104.058 ± 9.535 | | 98.470 ± 12.026 | | 9 5 ± 7.211 | | | 0.036* |
|  | Superior RNFL | 145.666 ± 1.154 | | | 126.882 ± 17.556 | | 123.411 ± 24.233 | | 120.625 ± 16.877 | | | 0.158 |
|  | Nasal RNFL | 75.666 ± 19.857 | | | 72.941 ± 20.920 | | 68.176 ± 16.210 | | 66.375 ± 6.300 | | | 0.879 |
|  | Inferior RNFL | 142.666 ± 13.576 | | | 136.823 ± 21.923 | | 125.529 ± 22.187 | | 117.375 ± 17.565 | | | 0.133 |
|  | Temporal RNFL | 87.333 ± 7.571 | | | 79.470 ± 9.267 | | 77.058 ± 12.881 | | 75.500 ± 9.071 | | | 0.323 |
|  | Superior GCIPL | 100.000 ± 8.544 | | | 103.176 ± 9.146 | | 100.058 ± 8.692 | | 105.750 ± 8.762 | | | 0.366 |
|  | Inferior GCIPL | 106.000 ± 3.464 | | | 101.058 ± 6.721 | | 102.647 ± 9.656 | | 104.000 ± 9.380 | | | 0.691 |
|  | GCIPL Average | 103.000 ± 5.894 | | | 102.117 ± 6.847 | | 101.352 ± 8.211 | | 104.875 ± 7.949 | | | 0.707 |
|  | RPCP Mean | 56.333 ± 1.154 | | | 53.941 ± 3.344 | | 52.470 ± 4.473 | | 51.750 ± 4.131 | | | 0.250 |
|  | RPCP Superior | 57.000 ± 0.000 | | | 56.411 ± 3.985 | | 54.117 ± 3.238 | | 54.625 ± 2.503 | | | 0.130 |
|  | RPCP Nasal | 53.333 ± 3.055 | | | 51.176 ± 3.924 | | 51.588 ± 6.134 | | 48.875 ± 4.580 | | | 0.423 |
|  | RPCP Inferior | 59.333 ± 1.527 | | | 56.176 ± 4.362 | | 54.529 ± 6.472 | | 53.625 ± 5.950 | | | 0.243 |
|  | RPCP Temporal | 55.000 ± 3.000 | | | 52.705 ± 3.636 | | 49.882 ± 5.699 | | 52.250 ± 3.807 | | | 0.272 |
|  | FAZ | 0.456 ± 0.049 | | | 0.341 ± 0.094 | | 0.375 ± 0.103 | | 0.462 ± 0.133 | | | 0.073 |
|  | Superficial VD | 34.666 ± 0.577 | | | 41.411 ± 3.336 | | 38.705 ± 4.209 | | 35.500 ± 3.585 | | | 0.001* |
|  | Deep VD | 30.000 ± 0.000 | | | 34.705 ± 4.579 | | 32.588 ± 4.359 | | 30.875 ± 2.799 | | | 0.109 |
|  |  | **Group comparison** | | | **P value (Mann Whitney U)** | |  | |  | | |  |
|  | RNFL Pairwise comparisons | No MS vs Lithium monotherapy | | | 0.080 | |  | |  | | |  |
|  |  | No MS vs Lithium + Valproate | | | 0.013* | |  | |  | | |  |
|  |  | Lithium monotherapy vs Valproate monotherapy | | | 0.215 | |  | |  | | |  |
|  |  | Valproate monotherapy vs Lithium + Valproate | | | 0.448 | |  | |  | | |  |
|  | Superficial VD pairwise comparisons | No MS vs Lithium monotherapy | | | 0.007* | |  | |  | | |  |
|  |  | No MS vs Lithium + Valproate | | | 0.302 | |  | |  | | |  |
|  |  | Lithium monotherapy vs Valproate monotherapy | | | 0.187 | |  | |  | | |  |
|  |  | Valproate monotherapy vs Lithium + Valproate | | | 0.019* | |  | |  | | |  |
| Kurt 2023 | **OCT measure** | **Lithium (n = 26)** **mean ± sd** | | | **Valproic acid (n = 41)** **mean ± sd** | | **Control (n = 49)** **mean ± sd** | | **F statistic (One way ANOVA)** | | | **P value** |
|  | Right RNFL | 90.42 ± 10.11 | | | 85.10 ± 12.80 | | 90.57 ± 8.18 | | 3.576 | | | .031* |
|  | Right cmt | 254.23 ± 23.56 | | | 248.88 ± 24.05 | | 247.10 ± 21.10 | | 0.848 | | | 0.431 |
|  | Right GCL | 75.96 ± 11.51 | | | 79.46 ± 5.84 | | 77.20 ± 10.38 | | 1.252 | | | 0.29 |
|  | Left RNFL | 90.85 ± 11.62 | | | 83.78 ± 11.17 | | 91.49 ± 8.11 | | 7.318 | | | .001* |
|  | Left cmt | 254.42 ± 21.83 | | | 249.27 ± 28.20 | | 246.92 ± 21.41 | | 0.824 | | | 0.441 |
|  | Left GCL | 75.23 ± 11.22 | | | 78.44 ± 8.99 | | 77.88 ± 9.76 | | 0.913 | | | 0.404 |
|  | **Gabriel test** | **Li-VPA**  **Mean difference ± sd, p-value** | | | **Li-Control Mean difference ± sd, p value** | | **VPA-Control Mean difference ± sd, p value** | | | | | |
|  | Right RNFL | 5.5.33 ± 2.62, P=.123 | | | −0.15 ± 2.53, P = 1.000 | | −5.47 ± 2.21, P = .043 | | | | | |
|  | Left RNFL | 7.07 ± 2.53,  P=.017 | | | −0.64 ± 2.45), P=.991 | | −7.71 ± 2.14, P=.01 | | | | | |
| Torun 2023 |  | **Bipolar lithium (n=39)** | | | **Healthy controls (n=36)** | |  | |  | | |  |
|  | Mean RNFL Thickness (µm) | 101,44±9,5  (86–122) | | | 100,06±9,5  (74–120) | | 0,532 | |  | | |  |
|  | CMT (µm) | 241,54±19,9  (207–290) | | | 267,61±20,2  (229–318) | | 0,0001** | |  | | |  |
|  |  |  | | |  | |  | |  | | |  |
| Gokcinar 2025 | **OCT measure** | **Bipolar lithium (n = 31),** µm**, mean ± sd** | | | **Bipolar valproate (n = 19), mean ± sd** | | **Mean difference** | | **P-value** | | |  |
|  | Inferior right eye | 131.16 ± 17.78 µm | | | 117.37 ± 17.06 µm | | 13.79 µm | | 0.009 | | |  |
|  | Inferior left eye | 117.74 ± 10.47 µm | | | 126.26 ± 18.62 µm | | 8.52 µm | | 0.044 | | |  |
|  | Peripapillary RNFL superior, nasal, temporal, average (left and right) | No evidence of statistically significant difference between the two groups, no figures provided. | | | | | | | | | | |
|  | Macular GCC (superior, inferior, average, left and right eyes) | No evidence of statistically significant difference between the two groups, no figures provided. | | | | | | | | | | |
| Egeli-Karatas (28) | **OCT Measure** | **Right Eye Li (mean±SD)** | **Right Eye SV-VPA (mean±SD)** | **Right Eye AP (mean±SD)** | **Right Eye p value** | **Left Eye Li (mean±SD)** | | **Left Eye SV-VPA (mean±SD)** | **Left Eye AP (mean±SD)** | **Left Eye p value** | | |
|  | RNFL Nasal superior | 115.11±24.69 | 110.19±22.81 | 108.07±20.18 | 0.473 | 127.36±25.57 | | 122.05±31.57 | 113.1±18.48 | 0.085 | | |
|  | RNFL Nasal | 82.80±18.71 | 78.75±15.73 | 75.85±14.30 | 0.276 | 75.11±13.77 | | 77.44±17.09 | 69.21±11.92 | 0.206 | | |
|  | RNFL Nasal Inferior | 122.52±27.52 | 113.66±29.93 | 115.64±27.85 | 0.044* | 122.69±25.96 | | 112.97±35.78 | 115.14±22.15 | 0.166 | | |
|  | RNFL Temporal superior | 143.86±23.44 | 137.50±24.31 | 135.75±14.84 | 0.09 | 139.61±18.07 | | 135.83±36.44 | 138.03±16.0 | 0.464 | | |
|  | RNFL Temporal | 72.86±12.56 | 72.08±10.37 | 70.85±8.86 | 0.438 | 72.16±15.51 | | 73.52±14.27 | 68.89±8.61 | 0.627 | | |
|  | RNFL Temporal Inferior | 154.75±21.27 | 141.91±21.38 | 143.64±17.86 | 0.008* | 152.11±23.14 | | 143.88±34.04 | 143.6±17.65 | 0.093 | | |
|  | RNFL Mean | 106.02±11.40 | 100.5±11.56 | 99.53±9.34 | 0.002* | 105.19±10.22 | | 100.16±18.77 | 98.17±9.15 | 0.004* | | |
|  | GCL | 1.17±0.07 | 1.11±0.12 | 1.10±0.08 | 0.002* | 1.17±0.07 | | 1.12±0.08 | 1.10±0.08 | 0.005* | | |
|  | IPL | 0.98±0.19 | 0.90±0.18 | 0.91±0.05 | 0.009* | 0.96±0.07 | | 0.91±0.06 | 0.91±0.05 | <0.001* | | |

OCT, Optical coherence tomograohy; GCL, ganglion cell layer; RNFL, retinal nerve fiber layer; sd, standard deviation; μm, micrometre; mm, millimetre; RNFLT, RNFL thickness; GCC, Ganglion cell complex; GCIPL, Ganglion cell Inner Plexiform Layer; IPL, Inner plexiform layer; RPCP, Radial peripapillary capillary plexus; FAZ, foveal avascular zone; VD, vessel density; CMT, centrale macular thickness; MS, mood stabiliser; n, number

Table 2: Summary of data and statistics for individual studies measuring functional outcomes of the retina

| Paper | Results | | | | | | | |  | | | |
| --- | --- | --- | --- | --- | --- | --- | --- | --- | --- | --- | --- | --- |
| Ullrich 1985 (31) | **DAT outcomes** |  |  |  | | | |  | | | | |
|  | Adaptation (n = 3) | No numerical figures reported |  |  | | | |  | | | | |
|  | **EOG** |  |  |  | | | |  | | | |  |
|  | **Arden ration (mean left and right eye)** | **L0: Pretreatment values** | **L1: 4^th^/5^th^ day of lithium** | **L2: 9^th^/10^th^ day of lithium** | | | | **L01: 14 days after lithium stopped** | | | |  |
|  | Participant 1 | 187 | 204 | 164.5 | | | | 209.5 | | | |  |
|  | Participant 2 | 198.5 | 170 | 155.5 | | | | 204.5 | | | |  |
|  | Participant 3 | 135.5 | 127.5 | 131.5 | | | | **-** | | | |  |
|  | Participant 4 | 337.5 | 252.5 | 258.5 | | | | 313 | | | |  |
|  | Participant 5 | 175.5 | 169.6 | 171.5 | | | | 146.5 | | | |  |
|  | Participant 6 | 249 | 262.5 | 265 | | | | 306 | | | |  |
|  | Participant 7 | 166 | 139 | 163 | | | | 180 | | | |  |
|  | Participant 8 | 230 | 246 | 217.5 | | | | 229.5 | | | |  |
|  | Mean | 210 | 196.4 | 190.9 | | | | 227 | | | |  |
|  |  |  | 5% reduction from baseline (p < 0.05)  Wilcoxon-test, one-tailed) | 10% reduction from baseline (p < 0.05)  Wilcoxon-test, one-tailed) | | | | Recovery significant at the 5% level | | | |  |
|  | **ERG outcome** | | | | | | | | | | | |
|  | Scotopic and photopic a and b wave analysis | No numerical figures reported | | | | | | | | | | |
| Carney 1988 | **DAT Measure** | **Patients (Std. Dev.)** | **Controls (Std. Dev.)** | | **p** | **Statistic** | |  | | |  | |
|  | First Descending Threshold: | 3.424 (0.511) | 3.191 (0.412) | | 0.132 |  | |  | | |  | |
|  | min. 10 | 2.296 (0.381) | 2.013 (0.232) | | 0.01 | Repeated measure ANOVA | |  | | |  | |
|  | min. 15 | 2.040 (0.402) | 1.722 (0.209) | | 0.005 |  |  |  | | |  | |
|  | min. 20 | 1.914 (0.361) | 1.633 (0.220) | | 0.007 |  |  |  | | |  | |
|  | min. 25 | 1.861 (0.355) | 1.548 (0.214) | | 0.003 |  |  |  | | |  | |
|  | min. 30 | 1.825 (0.367) | 1.472 (0.254) | | 0.002 |  |  |  | | |  | |
|  | Finishing level | 1.872 (0.357) | 1.562 (0.218) | | 0.003 |  |  |  | | |  | |
|  | **DAT Measure** | **Group comparison** | **Result** | | **P value** | **Statistic** | |  | | |  | |
|  | Cone portion (Minutes 1-9) | Male patients, male controls | Higher threshold for male patients and male controls | | p <0.05 | ANOVA | |  | | |  | |
|  | Rod portion (Minutes 10-30) | Male patients, male controls, female patients | Male patients higher threshold than male controls | | p <0.01 | ANOVA | |  | | |  | |
|  |  |  | Male patients higher threshold than female patients | | p< 0.05 | ANOVA | |  | | |  | |
|  | First Descending Threshold | Patients with family history of psychiatric illness other than affective disorder, unclear comparison group |  | | p <0.05 |  | |  | | |  | |
|  | Finishing threshold |  |  | | p < 0.02 |  | |  | | |  | |
|  | Correlation between number of episodes and first descending threshold | Patient group | Positive | | p < 0.05 |  | |  | | |  | |
| Kaschka 1988 (32) | **DAT measure** |  |  | |  |  | |  | | |  | |
|  | Dark Adaptation (n = 16) | No numerical figures reported |  | |  |  | |  | | |  | |
|  | **EOG measure** |  |  | |  |  | |  | | |  | |
|  | Arden ratio (average of 6 repeats) | Without lithium RE | With lithium RE | | p value RE | Without lithium LE | | With lithium LE | | | p value LE | |
|  | Participant 1 | 3.18 ± 0.98 | 1.69 ± 0.26 | | < 0.05 | 2.92 ± 0.94 | | 1.59 ± 0.22 | | | < 0.05 | |
|  | Participant 2 | 3.30 ± 0.46 | 1.96 ± 0.40 | | < 0.002 | 3.11 ± 0.30 | | 1.94 ± 0.43 | | | < 0.01 | |
|  | Participant 3 | 2.54 ± 0.53 | 1.92 ± 0.69 | | n.s. | 2.55 ± 0.52 | | 2.67 ± 0.12 | | | n.s. | |
|  | Participant 4 | 3.08 ± 0.61 | 2.78 ± 0.71 | | n.s. | 2.64 ± 0.36 | | 2.81 ± 0.55 | | | n.s. | |
|  | Participant 5 | 5.51 ± 2.31 | 3.74 ± 0.83 | | < 0.01 | 5.16 ± 2.45 | | 3.59 ± 0.70 | | | < 0.25 | |
|  | Participant 6 | 3.71 ± 0.96; | 2.17 ± 0.20 | | < 0.02 | 4.29 ± 1.19 | | 2.34 ± 0.24 | | | < 0.02 | |
|  | Participant 7 | 2.49 ± 0.66 | 1.61 ± 0.07 | | < 0.1 | 2.52 ± 0.65 | | 1.60 ± 0.11 | | | < 0.005 | |
|  | Participant 8 | 2.33 ± 0.29 | 2.01 ± 0.18 | | < 0.05 | 2.30 ± 0.22 | | 2.07 ± 0.19 | | | < 0.1 | |
|  | Participant 9 | 2.00 ± 0.39 | 1.81 ± 0.41 | | n.s. | 2.16 ± 0.63 | | 1.87 ± 0.34 | | | n.s. | |
|  | Participant 10 | 2.54 ± 0.36 | 2.47 ± 0.29 | | n. s. | 2.51 ± 0.36 | | 2.61 ± 0.35 | | | n.s. | |
|  | Participant 11 | 2.44 ± 0.48 | 1.91 ±0.22 | | <0.05 | 2.17 ±0.34 | | 1.68 ±0.16 | | | <0.01 | |
|  | Participant 12 | 2.49 ± 0.46 | 1.65 ±0.15 | | <0.005 | 2.45 ± 0.36 | | 1.57 ±0.24 | | | <0.005 | |
|  | Participant 13 | 2.93 ± 0.41 | 2.75 ± 0.43 | | n.s. | 2.60 ± 0.35 | | 2.87 ± 0.68 | | | n.s. | |
|  | Participant 14 | 2.28 ± 0.51 | 1.68 ±0.19 | | <0.05 | 1.74 ±0.15 | | 1.53 ±0.19 | | | <0.1 | |
|  | Participant 15 | 2.88 ± 0.45 | 2.78 ± 0.64 | | n.s. | 2.73 ± 0.43 | | 2.61 ± 0.42 | | | n.s. | |
|  | Participant 16 | 2.45 ± 0.26 | 2.80 ± 0.70 | | n.s. | 2.62 ±0.19 | | 2.77 ± 0.54 | | | n.s. | |
|  | Participant 17 | 3.31 ± 0.69 | 2.59 ± 0.70 | | n. s. | 3.01 ± 0.89 | | 2.45 ± 0.45 | | | n. s. | |
|  | Participant 18 | 2.85 ± 0.44 | 2.04 ±0.12 | | <0.05 | 2.97 ± 0.31 | | 2.08 ± 0.26 | | | <0.05 | |
|  | Participant 19 | 2.20 ± 0.62 | 1.83 ±0.21 | | n.s. | 2.17 ± 0.60 | | 1.91 ±0.24 | | | n.s. | |
|  | Mean values | 2.87 | 2.22 | |  | 2.77 | | 2.24 | | |  | |
| Seggie 1988 | **DAT** | Comparison groups | | Statistic | | | P value |  | | | | |
|  |  | Unmedicated patient vs healthy control in first 5 minutes | Patient demonstrated marked sensitivity to light | F = 15.6, df 1, 28 | | | p < .001 |  | | | | |
|  |  | Patient on lithium vs healthy control 6-10 minutes | Minimal detectable intensity of the patient when on lithium was significantly less than control values | F = 84.1, df 1, 28, | | | p < .001 |  | | | | |
| Seggie 1989 | **DAT** |  |  |  | | |  |  | | | | |
|  | Controls decrease in threshold over time for rod response | F = 250, df 8, 136 | < 0.001 | **No significant effect due to sex or a sex x time interaction** | | |  |  | | | | |
|  | Controls decrease in threshold over time for rod response | F = 32.3, df 4, 68 | < 0.001 | **No significant effect due to sex or a sex x time interaction** | | |  |  | | | | |
|  | Bipolar participants decrease in threshold over time for cone response | F = 186, df 8, 136 | < 0.001 |  | | |  |  | | | | |
|  | Bipolar participants decrease in threshold over time for rod response | F = 36.1, df 4, 68 | < 0.001 | Significant sex effect (F = 4.69, df 1, 17, p = 0.45), with male patients demonstrating a higher threshold than female patients | | |  |  | | | | |
|  | Bipolar females vs control females | No significant differences between thresholds and no group x time interactions | |  | | |  |  | | | | |
|  | Bipolar males vs control males cone portion (Minutes 1-9) | F = 4.78, fd, 1, 22 | p = .04  (Male controls significantly lower thresholds than male patients) | Significant interaction of group x time (F = 2.56, df 8, 176, p = 0.012) | | |  |  | | | | |
|  | Bipolar males vs control males rod portion (Minutes 10-30) | F = 28.8, df 1, 11 | < .001  (Male controls had significantly lower thresholds than male patients) |  | | |  |  | | | | |
| Emrich et al 1990 | **DAT measure** | **Healthy controls before lithium (n = 15)** | **Healthy controls after lithium (n = 15)** | **Healthy controls after lithium discontinuation (n = 15)** | | | **Affective psychoses taking lithium (n = 30)** | **Statistics** | | | | |
|  | DAT at 20 minutes | 0.50 to 0.80 log units (mean±SD=0.59±0.13) | 0.02 to 0.40 log units (mean±SD=0.21±0.13) | No figure provided | | | **0.02 to 0.50 log units (mean±SD=0.20±0.12)** | Before and after lithium healthy controls: Wilcoxon test, two tailed, p = 0.01  Lithium and postlithium healthy controls: Wilkoxon test, two-tailed, p = 0.001  ANOVA all three groups healthy controls: Wilks lambda = 0.05, approximate F = 117.61, df = 2, 13, p<0.00005  No statistically significant difference between affective psychoses taking lithium and healthy controls taking lithium (no figures provided) | | | | |
|  |  | **Affective psychoses subgroup (n = 7) before lithium** | **Affective psychoses subgroup (n = 7) after lithium** | **Statistics** | | | | | | | | |
|  | DAT at 20 minutes | 0.50 to 0.80 log units (mean±SD=0.59±0.13) | 0.02 to 0.40 log units (mean±SD=0.21±0.13). | Wilcoxon test, two tailed, p<0.001) | | | | | | | | |
| Schmidt-Betschel 1994 | EOG Measure | Patients (20 eyes) | Controls | P value | | | |  | | | | |
|  | Baseline potential | 0,408 ± 0,085 mV | 0,341 ± 0,104 mV | 0.01486 | | | |  | | | | |
|  | Light increase | 0,745 ± 0,182 mV | 0,676 ± 0, 173 mV | n.s. | | | |  | | | | |
|  | Light/dark coefficient | 1,82 ± 0,23 | 2,03 ± 0,23 | 0.00175 | | | |  | | | | |
| Lam 1997 | Healthy controls vs euthymic participants taking long term lithium | ERG b-wave and implicit times | EOG ratio | Dark and light adapted EOG amplitudes | | | |  | | | | |
|  | Repeated measures ANOVA for eye | (F = 1.2, df = 1,43, p > .20) | (F = 0. 1, df = 1,43, p > .70) | No significant effect (no statistic available) | | | |  | | | | |
|  | Repeated measures ANOVA for diagnosis | (F = 0.6, df = 1,43, p > .40) | (F = 0.02, df = 1,43, p > .80) | No significant effect (no statistic available) | | | |  | | | | |
|  | Repeated measures ANOVA by eye interaction effect | (F = 0.3, df = 1,43, p > .60) | (F = 0.1, df = 1,43, p > .70 | No significant effect (no statistic available) | | | |  | | | | |
| Wirz-Justice 1997 | **DAT measure** | **Bipolar disorder or schizoaffective disorder - taking lithium (n = 67)** | **Age matched controls (n = 33)** | **F statistic** | | | | **P value** | | | | |
|  | Cone adaptation Threshold | 5.85 _+ 0.61 log units | 6.29 -+ 0.28 (SD) log units | 2 | | | | <0.0001 | | | | |
|  | Rod adaptation threshold | 2.59 + 0.46 log units; | 2.27 _+ 0.30 log units | 7.28 | | | | <0.008 | | | | |
|  | **EOG measure** | **Bipolar disorder or schizoaffective disorder - taking lithium and other medication (n = 72)** | **Bipolar disorder or schizoaffective disorder - taking lithium only (n = 8 eyes)** | **Age matched controls (n = 8 eyes)** | | | | | | | | |
|  | Arden Index | 1.75 -+ 0.31 | 1.7 _+ 0.14 | 1.89 _+ 0.25 | | | | | | | | |
|  | **ERG measure** | Bipolar disorder or schizoaffective disorder - taking lithium (n = 38-40) | Controls (prior established value, n = unknown) | | | | | | | | | |
|  | ERG - b wave latency (scotopic) | 86.3 +_ 7.8 msec (right eye) 85.3 _+ 6.3 msec (left eye)  N = 38-40 | 76.1-94.8 msec | | | | | | | | | |
|  | ERG - b wave amplitude  (scotopic) | 154.6 _+ 67.5 ~V (right eye) 161.3 _+ 50.9 I.tV (left eye)  N = 38-40 | 142-402 ~V | | | | | | | | | |
|  | ERG - b wave latency (photopic) | 91.73 _+ 2.27 msec (right eye)  91.86 _+ 2.62 (left eye)  N = 25 |  | | | | | | | | | |
|  | ERG - b wave amplitude  (photopic) | 112.70 _+ 29.81 ~V (right eye)  106.3 -4- 30.36 I~V (left eye)  N = 25 |  | | | | | | | | | |
| Madsen 2021 | PIPR | 31 bipolar disorder participants, 12 of whom take lithium | Linear regression model | No association between lithium use and PIPRlate | | | |  | |  | | |

DAT, Dark Adaptation Therapy; EOG, electrooculogram; ERG, electroretinogram; n, number; PIPR, post illumination pupillary response
